# Therapeutic Potential of Sodium Channel Blockers as a Targeted Therapy Approach in *KCNA1*-Associated Episodic Ataxia and a Comprehensive Review of the Literature

**DOI:** 10.3389/fneur.2021.703970

**Published:** 2021-09-09

**Authors:** Stephan Lauxmann, Lukas Sonnenberg, Nils A. Koch, Christian Bosselmann, Natalie Winter, Niklas Schwarz, Thomas V. Wuttke, Ulrike B. S. Hedrich, Yuanyuan Liu, Holger Lerche, Jan Benda, Josua Kegele

**Affiliations:** ^1^Department of Neurology and Epileptology, Hertie Institute for Clinical Brain Research, University of Tübingen, Tübingen, Germany; ^2^Institute of Neurobiology, University of Tübingen, Tübingen, Germany; ^3^Bernstein Center for Computational Neuroscience Tübingen, Tübingen, Germany; ^4^Department of Neurosurgery, University of Tübingen, Tübingen, Germany

**Keywords:** episodic ataxia, sodium channel blockers, K_V_1.1, conduction-based model, voltage-gated potassium channels, riluzole, precision medicine, *KCNA1*

## Abstract

**Introduction:** Among genetic paroxysmal movement disorders, variants in ion channel coding genes constitute a major subgroup. Loss-of-function (LOF) variants in *KCNA1*, the gene coding for K_V_1.1 channels, are associated with episodic ataxia type 1 (EA1), characterized by seconds to minutes-lasting attacks including gait incoordination, limb ataxia, truncal instability, dysarthria, nystagmus, tremor, and occasionally seizures, but also persistent neuromuscular symptoms like myokymia or neuromyotonia. Standard treatment has not yet been developed, and different treatment efforts need to be systematically evaluated.

**Objective and Methods:** Personalized therapeutic regimens tailored to disease-causing pathophysiological mechanisms may offer the specificity required to overcome limitations in therapy. Toward this aim, we (i) reviewed all available clinical reports on treatment response and functional consequences of *KCNA1* variants causing EA1, (ii) examined the potential effects on neuronal excitability of all variants using a single compartment conductance-based model and set out to assess the potential of two sodium channel blockers (SCBs: carbamazepine and riluzole) to restore the identified underlying pathophysiological effects of K_V_1.1 channels, and (iii) provide a comprehensive review of the literature considering all types of episodic ataxia.

**Results:** Reviewing the treatment efforts of EA1 patients revealed moderate response to acetazolamide and exhibited the strength of SCBs, especially carbamazepine, in the treatment of EA1 patients. Biophysical dysfunction of K_V_1.1 channels is typically based on depolarizing shifts of steady-state activation, leading to an LOF of *KCNA1* variant channels. Our model predicts a lowered rheobase and an increase of the firing rate on a neuronal level. The estimated concentration dependent effects of carbamazepine and riluzole could partially restore the altered gating properties of dysfunctional variant channels.

**Conclusion:** These data strengthen the potential of SCBs to contribute to functional compensation of dysfunctional K_V_1.1 channels. We propose riluzole as a new drug repurposing candidate and highlight the role of personalized approaches to develop standard care for EA1 patients. These results could have implications for clinical practice in future and highlight the need for the development of individualized and targeted therapies for episodic ataxia and genetic paroxysmal disorders in general.

## Introduction

**Figure 1:**
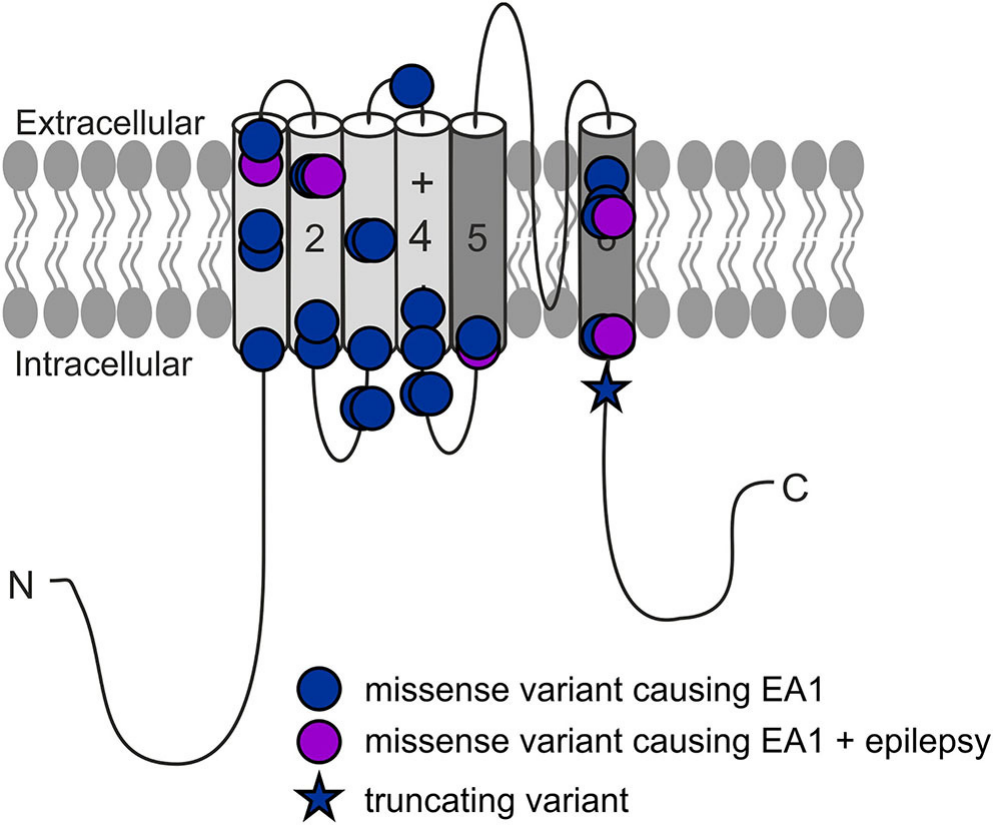
All reported *KCNA1* variants with EA1 phenotype.

Episodic ataxia type 1 (EA1) is an autosomal dominant ion channel disorder mainly caused by missense variants in the *KCNA1* gene on chromosome 12 (1) encoding the α-subunit of the voltage-gated potassium channel K_V_1.1 (Figure 1), which are expressed in the peripheral and the central nervous system including cerebellum and mostly present as heterotetramers with K_V_1.2 and K_V_1.4 subunits (2). It has been shown previously that loss-of-function (LOF) variants cause neuronal hyperexcitability due to disturbed repolarization and subsequently prolonged duration of action potentials [APs; (3)]. Finally, these biophysical changes clinically result in typical frequent and short-lasting attacks (seconds to minutes) which are characterized by gait incoordination, limb ataxia, truncal instability, dysarthria, nystagmus, tremor, occasionally seizures [around 10%; (4, 5); overview in (6)], but also persistent neuromuscular symptoms like myokymia or neuromyotonia (7). Patients often experience multiple attacks per day, some of which may cluster (5) and can have preceding sensory warning symptoms (8, 9). Common triggers for the paroxysmal events are startle reactions, vigorous activity, change in posture (e.g., from sitting to standing), emotion, hunger, alcohol, or intercurrent illness. The age of onset of EA1 attacks is usually in early childhood or adolescence [almost all below 20 years, on average 7.8 years; (7)] and attacks can abate in adulthood (8, 9). Cerebellar function is typically normal between attacks, but persistent cerebellar features have also been described (7) and disease course in general does not seem to follow a progressive mode (10). Brain imaging in patients with EA1 is typically unremarkable but may reveal cerebellar atrophy [around 10% of cases; (7)].

**Figure 2:**
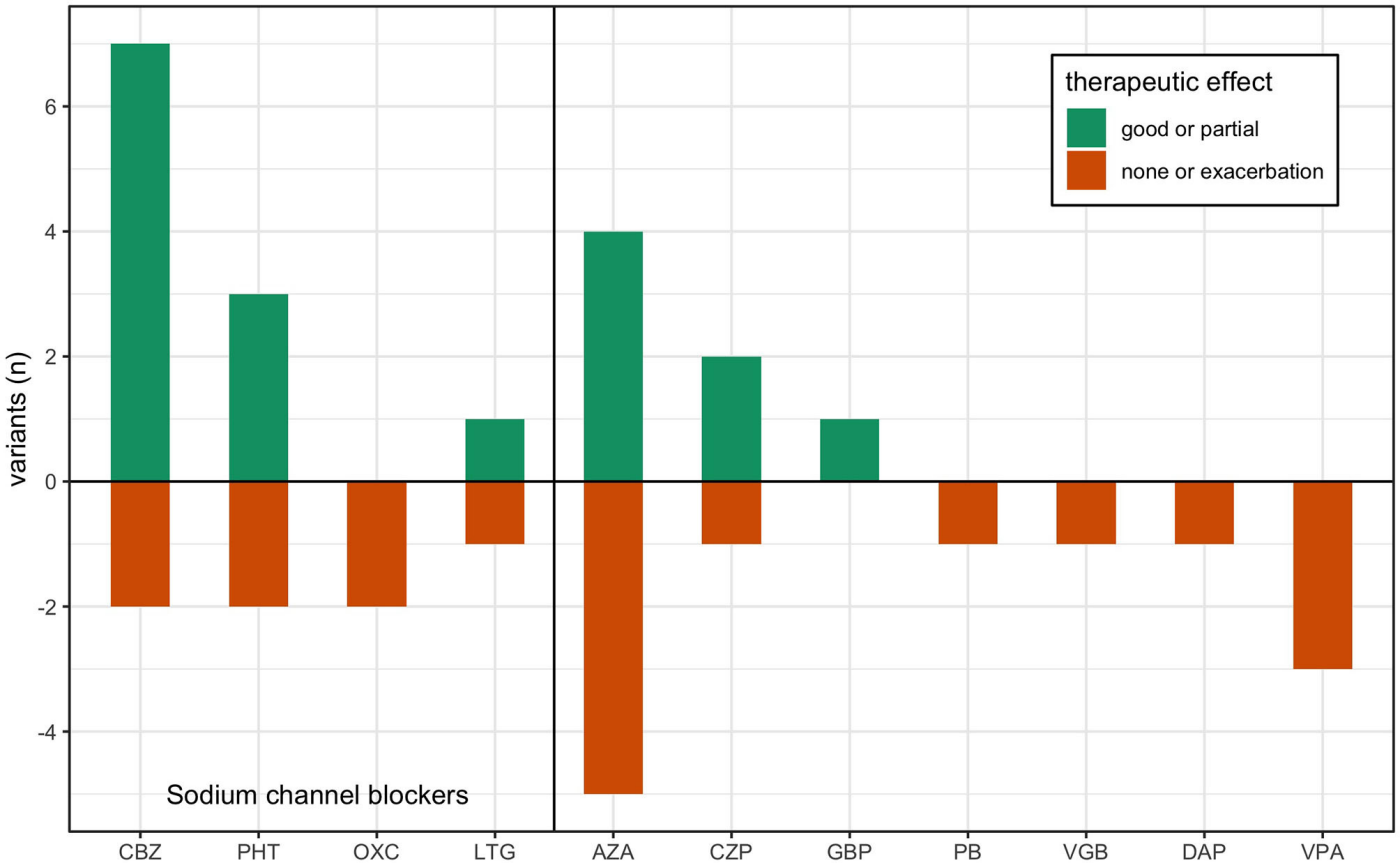
Treatment response in previous reports of *KCNA1* variants. Data is adapted from Table 1, where previous single case reports of EA1 are grouped by their respective disease-causing variant. Treatment responses are reported ranked by good effect (dark green), partial or varied effect (light green), and lack of effect or exacerbation (red) per the author's assessment. AZA, acetazolamide; CBZ, carbamazepine; PHT, phenytoin; PB, phenobarbital; VPA, valproic acid; LTG, lamotrigine; GBP, gabapentin; CZP, clonazepam; DAP, 3,4-diaminopyridine; OXC, oxcarbazepine; VGB, vigabatrin.

Already in 1994 “point mutations” in *KCNA1* were associated with EA1 (1), but the majority of variants and an ever-growing number of *de novo* variants have been identified in the last decade by new (and affordable) technologies such as next generation sequencing providing the basis for subsequent pathophysiological studies (summary in Table 1). At the same time, the unfolding complexity of the pathophysiological landscape of genetic paroxysmal movement disorders, such as episodic ataxias, emerges as one likely reason for limited therapeutic success experienced with a variety of therapeutic approaches, demonstrating the need for standards in the care of this increasing group. While a small subset of individuals with EA1 caused by LOF variants in *KCNA1* has shown quite good therapeutic response to the commonly used carbonic anhydrase inhibitor acetazolamide (AZA), five of nine attempted treatments were not successful (see Table 1; Figure 2). Although intuitive personalized therapeutic regimens tailored to disease-causing pathophysiological mechanisms may offer the specificity required to overcome intractability, for example, by using an enhancer for voltage-gated potassium channels (11), they are currently not available in clinical standard care. Nevertheless, by following this precision medicine approach, sodium channel blockers (SCBs) seem to constitute a promising alternative providing a biophysical rationale by “narrowing” the otherwise broadened shape of APs (12) through enhancement of the inactivated state (13). In addition, some SCBs show substance-specific and distinct mechanisms, which could contribute to functional compensation of variant channels. Riluzole, for example, displays a reduction of the persistent sodium current (14) and additional direct effects on heterotetramers of voltage-gated potassium channels (15, 16), thereby owning additional potential to restore the loss of K_V_1.1 channel function. These results could have implications for clinical practice in the future and highlight the need for the development of individualized and targeted therapies for episodic ataxia and genetic paroxysmal disorders in general.

**Table 1:** Biophysical features and treatment response of all identified variants in *KCNA1* patients.

**Variant**	**Position**	**Phenotype**				**Treatment**	**Overall effect**	**Current amplitude (pA/pF)**		**V**_****1/2****_ **of Activation (mV)**		**Slope of activation (k)**		**Tau deactivation (ms)**		**References**
		**Ataxia**	**Neuromuscular**	**Seizures**	**Other features**			**WT**	**Mut**	**WT**	**Mut**	**WT**	**Mut**	**WT**	**Mut**	
R167M	S1	+	++				LOF	57.9	16.4	−32.8	−22.8					PMID:23349320
V174F	S1	+	+		Paroxysmal choreoathetosis	PHT ++ AZA – CBZ –	LOF	100	7.6	−28.8	6.3	8.1	6.1	22.8	26.0	PMID: 9526001
I177N	S1	+	+				LOF			−28.1	30.9	7.5	9.2	36.3	8.3	PMID: 12799903
F184C	S1	++	++	++	Tremor, visual disturbances	PHT +	LOF	4.1	1.6	−32	−4	10	10	5	11	PMID: 10383630
F184C							LOF	100	15.1	−28.8	−2.9	8.1	7.1	22.8	27.4	PMID: 9526001
C185W	S1	+	++		Migraine, hyperthermia, short sleep duration		LOF	57.9	17.3	−32.8	−28.4					PMID: 23349320
C185W							LOF			−28.9	−28.1	6.9	5.1			PMID: 25642194
T226A	S2	+	+				LOF	100	2.4	−28.8	−14.5	8.1	6	22.8	51.2	PMID: 9714564
T226M	S2	+	+				LOF	100	3.9	−28.8	−14	8.1		22.8	65.0	PMID: 9714564
T226R	S2	+	++	++	Cataplexy, sleep disturbances, hypercontracted posture, skeletal deformities	AZA ++ CBZ ++ PB – PHT – VPA –	LOF									PMID: 10414318^b^
R239S	S2	+	+				LOF	Total loss of function due to absence of current and reduced protein expression								PMID: 9526001
A242P	S2	+	+	++		LTG ++ AZA –	LOF	100	10	−27.1	−31.9	7.6	7.2	12.8	17.0	PMID: 11026449
F249C	S2-S3	+	+		Susceptibility to malignant hyperthermia		LOF	1	0.001							PMID: 27271339
F249I	S2-S3	+	+				LOF	100	1	−28.8	−29.4	8.1	6.2	22.8	44.7	PMID: 9526001
N255D	S3				Hypomagnesemia		LOF	398	72	−39	−8					PMID: 19307729
I262M	S3	+	++		Tremor, lower limb spasticity	GBP ++^a^ CZP ++^a^ VPA – PHT – 3,4-DAP –	LOF	186	14							PMID:24639406
I262T	S3	+	++		Distal muscle weakness and prolonged myotonia		LOF	1	0.3	−43.2	−9.6	4.4	9.4			PMID: 26778656
E283K	S3-S4	+	+		Mild deep sensory impairment, family history of diabetes	CBZ ++	LOF	114	76	−25.8	−16.5	7.4	7.8	22.4	30.8	PMID: 28666963
F303V	S4	+	+		Gaze-evoked nystagmus		LOF	14	0.5	−25	11	6	8			PMID: 28676720
R307C	S4	+	+		Headache, blurred vision, nausea, incoordination of hands	CBZ –^c^	LOF	86.8	22.1	−41.9	−30.5					PMID: 20660867
G311D	S4-S5	+	+		Diplopia during attacks	AZA – OXC – VPA –	LOF	60	20							PMID: 30140249
G311S	S4-S5	+					LOF	100	22.9	−28.8	2.9	8.1	13.4	22.8	20.7	PMID: 9714564
R324T	S4-S5	+	++	++	Suspected paroxysmal kinesigenic dyskinesia	CBZ ++ AZA –	LOF									PMID: 27477325^b^
E325D	S4-S5	+	+			AZA + CBZ +^d^	LOF	100	3.8	−28.8	31.6	8.1	17.5	22.8	3.5	PMID: 9526001
E325D							LOF			−18.3	3.5	8.3	12.6	32.8	52.7	PMID: 10428758
V404I	S6	+	+		Mild intellectual disability, motor developmental delay, short stature	CBZ ++ AZA ++	LOF			−27.1	−15.8	7.6	9.5	12.8	20.0	PMID: 11026449
I407M	S6	+	++		Blurred vision, hearing impairment		LOF	57.9	25.2	−32.8	11.4					PMID: 23349320
V408A	S6	+	+				LOF	4.1	2.7	−32	−31	10	10	17.6	0.8	PMID: 10383630
V408A							LOF	100	49.8	−28.8	−28.4	8.1	7.5	22.8	2.1	PMID: 9526001
V408A							LOF			−18.3	−17.4	8.3	10.2	32.8	29.2	PMID: 10428758
V408L	S6	++	++	+	Global developmental delay or mild intellectual disability, difficulty swallowing	PHT ++^e^	LOF	Total loss of function due to lack of protein expression								PMID: 19528245
F414C	C	+	+/++	+		CZP + AZA – OXC –	LOF			−26	−23.8	6	7.3			PMID: 18926884
F414S	C	+	+		Tremor, headache, visual disturbance, nausea, incoordination of hands, vertigo, dizziness	CBZ +	LOF	86.8	6.9	−41.9	−27					PMID: 20660867
R417Stop	C	++	+	++	Tremor	CBZ + AZA + LTG – VGB – CZP –	LOF	100	2	−27.1	−18.6	7.6	12.8	12.8	9.6	PMID: 11026449

*Clinical features and treatment responses are adapted and updated from D'Adamo et al. (ref), Paulhus et al. (ref) and Graves et al. (ref). Phenotypic features: Ataxia includes dysarthria and slurred speech, ranked as mild to moderate (+) or severe (++). Neuromuscular symptoms include myokymia (+) or more severe (neuro)myotonia and/or generalized stiffness (++). Seizures (+) or a diagnosis of epilepsy (++). Treatment response is ranked by good effect (++), partial or varied effect (+), and lack of effect or exacerbation (–). AZA, acetazolamide; CBZ, carbamazepine; PHT, phenytoin; PB, phenobarbital; VPA, valproic acid; LTG, lamotrigine; GBP, gabapentin; CZP, clonazepam; DAP, 3,4-diaminopyridine; OXC, oxcarbazepine; VGB, vigabatrin*.

a *This treatment was effective for muscle stiffness only*.

b *This variant has been functionally studied, but insufficient data is reported*.

c *CBZ was only tested in one patient and at a low dose (400 mg)*.

d *CBZ was only effective for potentially unrelated dystonic attacks*.

e *The authors note that PHT should be used with caution as it may contribute to cerebellar dysfunction or atrophy*.

Toward this aim, we (i) reviewed all available clinical reports on treatment response and functional consequences of all *KCNA1* variants causing EA1, (ii) examined the potential effects on neuronal excitability of all variants using a single compartment conductance-based model and set out to assess two SCBs (carbamazepine and riluzole) regarding their potential to restore the identified underlying pathophysiological mechanisms of *KCNA1* variant channels, and (iii) provide a comprehensive review of the literature considering all types of episodic ataxia.

## Materials and Methods

To study the potential effects of SCBs on neuronal firing behavior of *KCNA1* variants, we simulated the concentration dependent effects of carbamazepine and riluzole.

### Model Structure

We simulated neuronal firing behavior in a single-compartment conductance-based model of a pyramidal cell, based on Pospischil et al. (17). A cortical pyramidal neuron model was chosen as it is well-characterized, versatile, and these neurons are known to express K_V_1.1 channels (18, 19). The model cell has a cylindrical shape with length *L* and diameter *d* and its membrane equation

(1) $$\begin{array}{l} C_{m}\overset{∙}{V}=\;-\;I_{Na}\;-\;I_{K}\;-\;I_{K_{V}1.1}\;-\;I_{M}\;-I_{L}\;+\;I_{input} \end{array}$$

sums up a sodium current, *I*_*Na*_ = *g*_*Na*_*m*^3^*h* [*V* – *E*_*Na*_], a delayed rectifier potassium current, *I*_*K*_= *g*_*K*_*n*^4^ [*V* – *E*_*K*_], an K_V_1.1 current, *I*_*A*_ = *g*_*K*_*V*_1.1_*su* [*V* – *E*_*K*_], an M-type potassium current, *I*_*M*_ = *g*_*M*_*p* [*V* – *E*_*K*_], a leak current, *I*_*L*_ = *g*_*L*_[*V* – *E*_*L*_], the input current, *I*_*input*_, and the capacitive current with membrane capacitance *C* = 1μ*F/cm*^2^. The dynamics of the gating variables *m, h, n, p, s, u* of the ionic currents *I*_*i*_ with maximal conductance *g*_*i*_ and reversal potential *E*_*i*_ follow

(2) $$\begin{array}{l} {ẋ}_{i}=\;\alpha_{i}\left(V\right)\left[1-x_{i}\right]-\;\beta_{i}\left(V\right)x_{i} \end{array}$$

where ẋ_*i*_ is the derivative of the gating parameter *x*_*i*_ with respect to time, α_*i*_(*V*) are the opening rates and β_*i*_(*V*) the closing rates. Steady-state activation and inactivation curves as well as time constants are given by *x*_∞, *i*_(*V*) = α_*i*_(*V*)/[α_*i*_(*V*)+ β_*i*_(*V*)] and τ_*i*_(*V*) = 1/[α_*i*_(*V*)+ β_*i*_(*V*)], respectively.

Parameters for gating variables *m, h*, and *n* are taken from Traub and Miles (20); *p* is taken from Yamada (21); and *s* and *u* are taken from Ranjan et al. (22) and fitted to the mean wildtype biophysical parameters in Table 1. All parameters that differ from their original sources are g_Na_: 56 mS/cm^2^, g_K_: 5.4 mS/cm^2^, g_M_: 0.075 mS/cm^2^, g_K_V_1.1_: 0.6 mS/cm^2^, g_L_: 0.0205 mS/cm^2^, E_Na_: 50 mV, E_K_: − 90 mV, E_L_: − 70.3 mV, d = L: 61.4 μm, τ_max, p_: 608 ms.

To be able to apply changes as reported from electrophysiological characterizations of the kinetics of mutated ionic currents to the activation and inactivation properties, we fitted a sigmoid function

(3) $$\begin{array}{l} s_{i}\left(V\right)=\;\frac{1-a}{{\left[1+exp\left(-\left[V-V_{1\;/2}\right]/k_{i}\right)\right]}^{\gamma }}+a \end{array}$$

to the respective steady-state curves *x*_∞*, i*._ where *k*_*i*_ is the slope factor, *V*_1/2*, i*_ is the half-activation voltage, and γ an exponent to improve the accuracy of the fit. A persistent current could be added by changing *a*. The parameters for the different gates are summarized in Table 2.

**Table 2:** Gating parameters.

	***V*_**1/2**_ (*mV*)**	***k* (*mV*)**	**γ**	***a***
*m*	−34.3	−8.21	1.42	0
*h*	−34.5	4.04	1	0.05
*n*	−63.8	−13.8	7.35	0
*p*	−45.0	−10.0	1	0
*s*	−30.02	−7.73	1	0
*u*	−46.86	7.67	1	0.245

The model was integrated with the Euler forward method and time steps Δ*t* = 0.01 ms. All simulations and analysis were done with custom Python 3.8 software.

### *KCNA1* Variant Modeling

We simulated membrane responses to injected step currents ranging from 0 to 0.75 nA for 2 s and analyzed the voltage traces for their firing rate. The resulting f-I curve was then analyzed for its rheobase and its area under the curve (AUC) computed for the first 200 pA of steady-state firing.

The observed effects of each *KCNA1* variant on the biophysical properties of I_K_V_1.1_ activation (V_1/2_, slope, and current amplitude; Table 1) were implemented in the model K_V_1.1 current, and the effects of *KCNA1* variants on the firing behavior were simulated.

The effect of each of *KCNA1* variant *i* on the rheobase and AUC of the f-I curve was quantified relative to the unchanged wildtype model with

(4) $$\begin{array}{l} \Delta rheobase=\;rheobase_{i}\;-\;rheobase_{wt} \end{array}$$

(5) $$\begin{array}{l} AUC\;change=\;\frac{AUC_{i}\;-\;AUC_{wt}}{AUC_{wt}}\cdot 100\% \end{array}$$

The effects of SCBs carbamazepine and riluzole were modeled with alterations of current properties affected by the respective SCBs. For carbamazepine (CBZ), concentration dependent decreases in I_Na_ amplitude [1/(1 + [*CBZ*]/227 μ*M*); (23)] and hyperpolarizing shifts in I_Na_ inactivation [−20.5 *mV*/(1 + 116.4 μ*M*/[*CBZ*]); (24)] are modeled. These effects are in line with the effects seen at 100 μM in human hippocampal slices (25). The effects of 0, 5, 10, 15, 20, 25, 37.5, 50, 75, and 100 μM carbamazepine were simulated for each *KCNA1* variant.

Concentration dependent riluzole (RLZ) induced decreases in persistent I_Na_ amplitude [1 − 0.99365487/(1 + (2 μ*M*/[*RLZ*])^1.36^); (14)], I_Na_ amplitude [1 − [*RLZ*]^1.05^/(51 + [*RLZ*]^1.05^); (26)], I_K_ amplitude [(1 − [*RLZ*]/(88 μ*M* + [*RLZ*]); (26)] as well as concentration-dependent hyperpolarizing shifts in I_Na_ [$-24.84\;mV/\left(1+{\left(\frac{\left[RLZ\right]}{21.7}\mu M\right)}^{-1.11}\right)$; (14)] are simulated for each *KCNA1* variant from 0 to 30 μM in 5 μM increments.

For each *KCNA1* variant and the carbamazepine or riluzole concentration, firing behavior was quantified and compared to wildtype model firing with percentage change in AUC and Δrheobase.

### Search Strategy for Literature Review

MEDLINE, the Cochrane Library, conference abstracts, thesis and dissertations, preprint servers (bioRxiv, medRxiv), internet websites, and reference lists were searched in April 2021 to identify articles for inclusion. Only articles in English were considered. Keywords have been: “episodic ataxia” and all subtypes (EA 1-9), “*KCNA1*,” “K_V_1.1,” “*CACNA1A*,” “*CACNB4*,” “EAAT1,” “*SLC1A3*,” “*UBR4*,” “Na_V_1.2,” “*SCN2A*,” “*ATP1A3*,” “*PRRT2*,” “*SLC2A1*,” “*TBC1D24*,” “*KCNA2*,” “*CEP290*,” “*FGF14*,” “*NALCN*,” “paroxysmal movement disorders,” “functional consequences,” and “biophysical features.” Four reviewers independently screened literature search results and abstracted data from included studies. Descriptive analysis was conducted.

## Results

### Treatment Response and Functional Consequences of all EA1 Causing *KCNA1* Variants

In this study, we found 34 reports about 28 different variants in the *KCNA1* gene including electrophysiological data about their functional consequences. In 15 patients, 36 treatment efforts with 12 different drugs were described (see Table 1; Figure 2): 9 trials each with acetazolamide (AZA) and carbamazepine (CBZ), 5 trials with phenytoin (PHT), 3 trials with valproic acid (VPA), 2 trials with oxcarbazepine (OXC), lamotrigine (LTG), and clonazepam (CZP), and 1 trial with gabapentin (GBP), 3.4-diaminopyridine (DAP), vigabatrin (VGB), and phenobarbital (PB). Altogether, most positive results were seen for CBZ with seven reports of beneficial effects. One patient benefitted only in respect of potentially unrelated dystonic attacks and another non-responding patient was tested only with a low dose (400 mg/d). Effects of another SCB, PHT, were also remarkable, since three of five patients showed substantial clinical improvement. AZA showed mixed results with four patients exhibiting amelioration and five patients without positive effects. For all other drugs, only one or two reports are available, partially with unspecific positive clinical effects on muscle stiffness (see Table 1).

**Figure 3:**
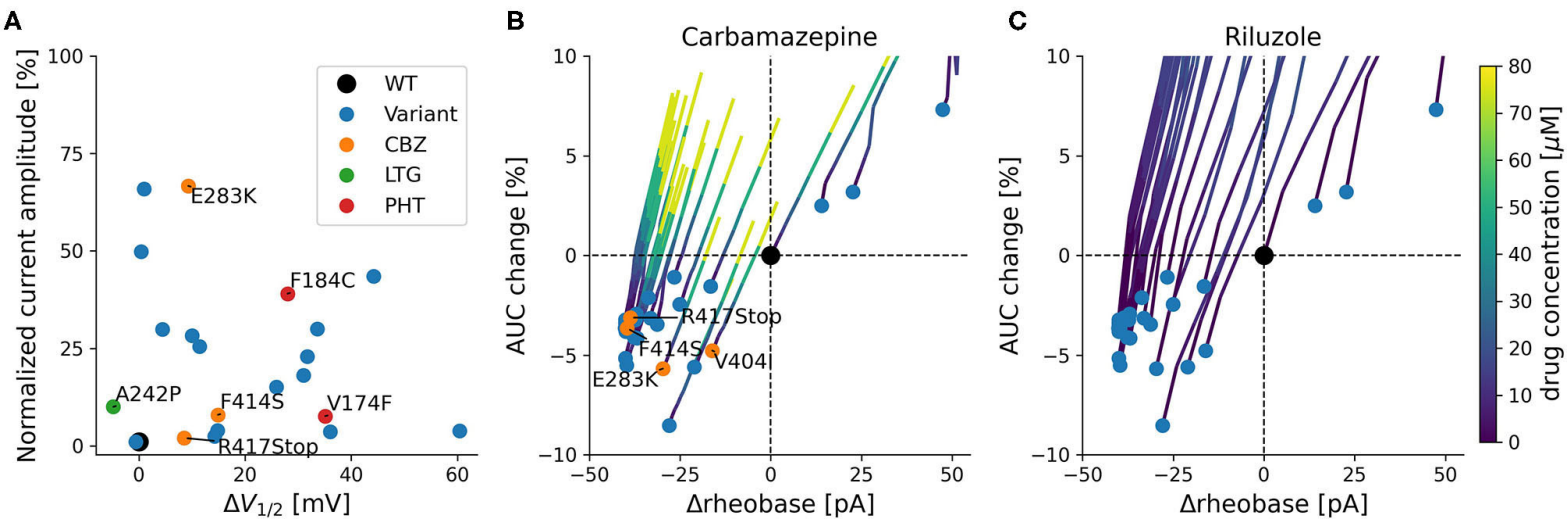
Functional effects of *KCNA1* variants with episodic ataxia 1 phenotype. **(A)** Current amplitudes and shifts of the variant's steady-state activation curves in relation to wildtype channels for all functionally characterized EA1 variants with available data. Positive treatment effects to SCBs are indicated (CBZ: orange, PHT: red, LTG: green). Selected variants are labeled with their respective mutation. **(B,C)** Simulated effects of variants on firing rheobase and change in AUC. Rheobase is measured relative to the wildtype (Δrheobase), and AUC change represents the percentage change relative to wildtype of the area under the curve of the fI-curve for the first 200 pA of steady-state firing. Blue dots depict available *KCNA1* variants. Orange dots represent patients that responded positively to CBZ (in B). Colored lines depict concentration dependent simulated changes in rheobase and AUC caused by CBZ **(B)** or RLZ **(C)**.

All variants with described ionic current kinetics showed distinct LOF behavior considering isolated K_V_1.1 function, which usually leads to hyperexcitability in a neuronal network. Common mechanisms of almost all of the 21 variants are reduced current amplitudes and depolarizing shifts of the steady-state activation curve (see Table 1; Figure 3). The only patient with a positive response to LTG (Figure 3A, green dot) had negative shift of the *KCNA1* activation curve. Positive responders to CBZ (Figure 3A, orange dots) showed a medium shift, and PHT responders (Figure 3A, red dots) showed a pronounced shift of the half activation voltage.

### Predicted Effects of Carbamazepine and Riluzole on *KCNA1* Variant Firing Behavior

Simulation-based estimates of *KCNA1* variant effects predict decreases in rheobase and relative mild decreases in firing rate quantified as the area under the curve of the f-I curve (AUC). These predictions were associated with increased neuronal excitability and were present in all but three *KCNA1* variants (Figures 3B,C).

The SCBs carbamazepine (CBZ) and riluzole (RLZ) both increased rheobase, consequently reducing neuronal excitability. In addition, they both counteracted the reduction in firing rate by increasing AUC (Figures 3B,C). The simulated responses to the drugs seem to be consistent over the different variants and have comparable slopes between CBZ and RLZ simulations, with RLZ being more sensitive to the respective drug concentration. The most effective drug in EA1 patients, carbamazepine, demonstrated positive treatment effects in *KCNA1* variants that have moderate changes in AUC and rheobase (F414S, V404I, R417Stop, and E283K). R307C variant channels exhibited also moderate AUC and rheobase changes, but the clinical reports did not show positive treatment effects with only low-dose carbamazepine (400 mg/day) in a limited dataset.

### Review of the Literature

#### Other Gene-Associated Episodic Ataxias

##### Episodic Ataxia Type 2

EA type 2 is not only the best-characterized but also represents the most common form of EAs, with an estimated prevalence of about 1/100,000 (10). Typical paroxysmal attacks are commonly longer than in EA1 (lasting hours to days) and are characterized by recurrent debilitating spells of unsteadiness, incoordination, vertigo, and dysarthria. Most patients with EA2 exhibit disease onset in early childhood; however, an overall wide range between 2 and up to 32 years has been reported. Frequency of episodes may vary from once or twice a year to several per week and may occur spontaneously or triggered by physical stress (frequently with first attacks while playing sports), fatigue, emotional distress, exercise, or drinks (i.e., alcohol and coffee). Interictal signs are common and may include slowly progressive ataxia and nystagmus [i.e., gaze-evoked, rebound, or primary position downbeat nystagmus, (27)]. Brain imaging may reveal cerebellar atrophy of the vermis. Acetazolamide is considered the drug of choice and may significantly reduce both frequency and severity of ataxic attacks (28). Additionally, 4-aminopyridine, a selective blocker for voltage-gated potassium channels (K_V_1 family) and FDA/EMA approved for symptomatic treatment of multiple sclerosis, has also been demonstrated to be effective in adolescent and adult individuals with EA2 (29).

**Figure 4:**
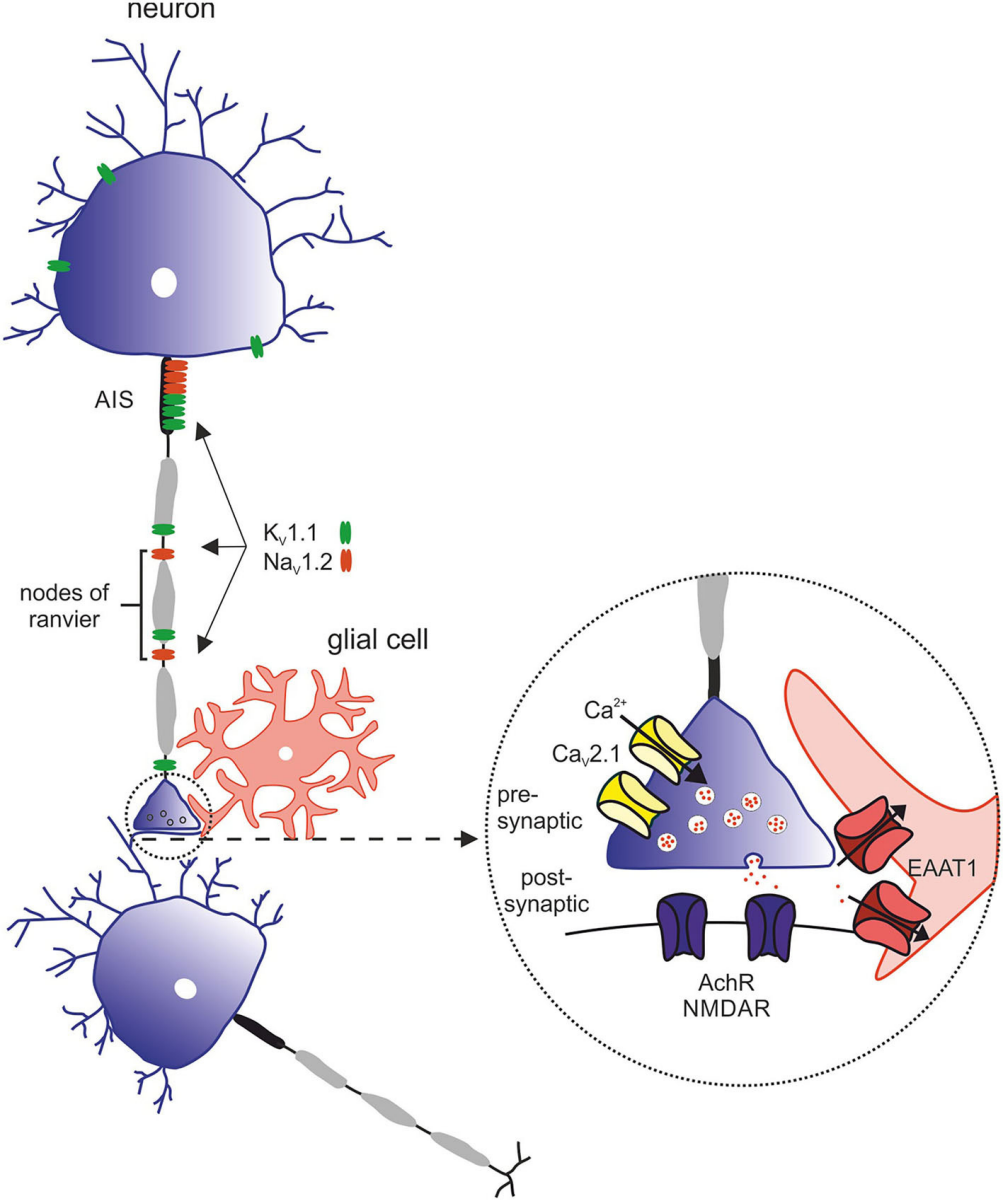
Overview of relevant ion channels and transporters in pathophysiology of episodic ataxia subtypes. Morphology of the neuron with its structural compartments—soma and dendrites (purple), axon initial segment (AIS, gray), and nodes of ranvier (black). Relevant ion channels of episodic ataxia subtypes: K_V_1.1 (green) → EA1, expressed in soma, AIS and nodes of ranvier. Na_V_1.2 (red) → EA9, predominantly expressed in the AIS and nodes of ranvier. Inset with representative synapse: Ca_V_2.1 (yellow) → EA2, presynaptic. EAAT1 (red) → EA6, postsynaptic. AchR and NMDAR (purple), postsynaptic.

EA2 is genetically determined by heterozygous variants in the *CACNA1A* gene, coding for the α1A subunit of P/Q type voltage-dependent calcium channels [Figure 4; (30)]. Electrophysiological studies mainly postulated LOF mechanisms underlying EA2 (31, 32), while in patients with familial hemiplegic migraine (FHM1) gain-of-function (GOF) variants have been identified. In contrast, mixed biophysical features (LOF and GOF) may account for epilepsy phenotypes (33). Of note, spino-cerebellar ataxia type 6 (SCA6) is caused by CAG triplet expansion in the same gene (34).

##### Episodic Ataxia Type 5

EA5 is associated with mutations in *CACNB4* (35), which encodes the voltage-dependent L-type calcium channel subunit β4 (Figure 4). The phenotypic spectrum cannot be determined well, since only very few individuals with EA5 have been described so far: By screening pedigrees with familial epilepsy and ataxia, Escayg et al. (35) identified the p.(482X) variant in an individual with juvenile myoclonic epilepsy without episodic ataxia and p.(Cys104Phe) in a family with episodic ataxia and another family with generalized epilepsy (GTCS and atypical absences) and “praxis-induced” seizures. In 2019, two further individuals (father and daughter) have been published with the same missense mutation [p.(Cys104Phe)], who suffered from mild adult onset episodic ataxia (36). Recently, two siblings have been reported with a complex neurodevelopmental disorder carrying a homozygous variant p.(Leu126Pro), though without episodic ataxia (37). Compared to EA2 patients, patients suffer from similar symptoms, but onset of manifestation is later in EA5. Long-term follow-up in one adult EA5 case has shown permanent ataxia. Brain imaging is typically unremarkable. Acetazolamide seems to be effective. Functional studies of the variants revealed alterations in the calcium channel function, such as decreasing time constants of variant channels leading to a decrease of net inward flow of calcium ions into activation. However, the authors state that functional alterations in the oocyte expression system are rather subtle and the pedigrees are too small to provide strong evidence for linkage (35). Identification of additional patients with mutations in *CACNB4* and EA phenotype failed, and designation remains contentious.

##### Episodic Ataxia Type 6

The first description of episodic Ataxia type 6 (EA6) was in 2005 in a 10-year-old boy with symptoms of EA as well as progressive ataxia, seizures, and migraine headaches with prolonged alternating hemiplegia triggered by mild head trauma and fever (38, 39). The phenotype could be linked to a *de novo* heterogenous missense mutation in *SLC1A3*, encoding the glial glutamate transporter EAAT1 (excitatory amino acid transporter 1, see Supplementary Figure 1). This mutation leads to an exchange of the highly preserved proline to arginine at position 290 [p.(Pro290Arg)]. Next to this mutation, four other mutations in *SLC1A3* could be associated with EA6: p.(Met128Arg) (40), p.(CysC186Ser) (41), p.(Val393Iso) (42), and p.(Arg499Glu) (43). All listed mutations are associated with clinical symptoms of EA as well as additional complaints such as migraine, tremor or slurred speech. Onset of disease ranged from 10 years [p.(Pro290Arg)] to 55 years [p.(Val393Iso)] (42). Symptoms partially responded to treatment with acetazolamide (39, 42). In addition, the mutation p.(Thr387Pro) was described in a case of a 22-year-old man with severe migraine headache accompanied by transient neurological deficits, including visual disturbances, prominent dysphasia, and unilateral sensory and motor deficits (44). The main purpose of the five EAAT subtypes is the reuptake of the excitatory neurotransmitter glutamate in the brain. In addition, they can work as anion channels (45, 46). In the latter function, the main glial glutamate transporter in the cerebellum, EAAT1, seems to play a crucial role in astrocytic anion homeostasis and volume regulation, especially in Bergmann Glia cells during glial maturation (47). In heterologous expression systems could be demonstrated that the mutation p.(Pro290Arg) impairs the glutamate transport rate, while increasing channel activity leading to an increased anion efflux (46, 48). This could be confirmed in a transgenic mouse model by Kovermann and others (46). The glutamate-activated chloride efflux in Bergmann glia triggered the apoptosis of these cells during infancy. The loss of Bergmann glia resulted in reduced glutamate uptake and impaired neural network formation in the cerebellar cortex (46). The migraine associated mutation p.(Thr387Pro) on the other hand diminishes hEAAT1 glutamate uptake rates by an altered ligand-binding to the transporter and reduces the number of hEAAT1 in the surface membrane (44). Functional studies of the other mutations mentioned are missing to date.

##### Episodic Ataxia Type 8

There has been a large non-consanguineous Irish family described whose members suffer from episodic ataxia beginning in the second year of life (49). Typical ictal features were unsteadiness, weakness, and dysarthria triggered by stress or tiredness. Compared to EA1-7, the onset of the EA in this family happens to be much earlier, muscular hypertrophy is absent, and there is no vertigo, tinnitus, limb, or neck jerking during the attacks. Minimal gaze-evoked nystagmus has been reported but no prominent eye movement disturbances. Interestingly, the affected individuals responded well to clonazepam, but acetazolamide was not effective. The disease has been linked to the genes *UBR4* [p.(Arg5091His)], which encodes an ubiquitine-protein ligand, and *HSPG2*, which encodes the structural protein perlecan. The authors suggested *UBR4* to be the more likely causative gene but did not exclude that *HSPG2* may also play a critical role in disease mechanism. The exact mechanism on how *UBR4* mutation may lead to the phenotype is not well-understood yet, although *UBR4* seems to play a role in calcium signaling (50). In a Korean study, where whole exome sequencing was performed in 39 individuals with episodic ataxia, four new possible or probable pathogenic variants in *UBR4* [p.(Tyr4877Cys), p.(Arg4111His), p.(Ala5042Val), and p.(Ala2581Val)] have been identified. Two of them [p.(Tyr4877Cys) and p.(Arg41111His)] co-occurred with variants in *CACNA1A* which is why the authors suggested that *UBR4* variants may have a modifier effect to *CACNA1A* (42).

##### Episodic Ataxia Type 9

Episodic ataxia type 9 (EA9) is caused by pathogenic variants in *SCN2A*, which encodes the α-subunit of the voltage-gated sodium channel Na_V_1.2. EA9 is characterized by an onset of ataxic episodes in the first years of life, which are usually preceded by tonic or generalized tonic-clonic seizures. The ataxic episodes have a broad phenotypic spectrum including walking difficulties, headache, vomiting, pain, slurred speech, and “dizziness” (51, 52). Symptoms manifest between the first months and 14 years of life and last from minutes to several weeks. The frequency varies between daily and one episode a year. The development of the patients ranges from slight retardation or mild autistic features to normal psychomotor development. Standard pharmaceutical treatment with acetazolamide was effective in half of the patients (53).

So far, two mutation hotspots have been identified. One of these is the mutation p.(Ala263Val), while the other includes the S4 segment and its cytoplasmic loop in domain IV of the channel (54). Electrophysiological studies showed a profound gain-of-function with a 3-fold increased persistent sodium current by the p.(Ala263Val) variant (51). Further investigations also revealed GOF effects by a hyperpolarizing shift of the activation curve [p.(Arg1882Gly)] or by increased current density along with a depolarizing shift of the steady-state inactivation curve [p.(Arg1882Gly) in combination with p.(Gly1522Ala)] (54). All these findings are consistent with neuronal hyperexcitability.

#### Other Gene-Associated Episodic Ataxias Based on Single Patients or Families

In contrast to the aforementioned types of episodic ataxia the following genes are associated with episodic ataxia, but not OMIM listed as distinct groups: *ATP1A3* (55), *PRRT2* (56), *SLC2A1* (57), *TBC1D24* (58), *KCNA2* (59), *CEP290* (60), *FGF14* (61), and *NALCN* (62).

#### Episodic Ataxias Associated With Mapped Disease Loci

Episodic ataxia types 3, 4, and 7 have not yet been linked to specific genes or variants. Most descriptions are based on single families with phenotypes that differ from EA1 and EA2 in conjunction with unremarkable screening of variants in *KCNA1* and *CACNA1A*.

##### Episodic Ataxia Type 3

Following the descriptions of EA1 and EA2, Steckley et al. (63) broadened the phenotypic spectrum of EAs by reporting a Canadian family of Mennonite heritage with vestibular ataxia, vertigo, tinnitus, and interictal myokymia. Interictal nystagmus was not present and episodes were shorter distinguishing this family from EA2 patients. Although sharing some common features with EA4 such as vertigo and tinnitus, the absence of other features (see section Episodic Ataxia Type 4) justifies this entity: Patients did respond to treatment with acetazolamide. Linkage analysis mapped the disease locus to a 4-cM region on 1q42; a causative mutation has not yet been identified (64).

##### Episodic Ataxia Type 4

EA4, also known as periodic vestibulocerebellar ataxia (PATX) or North Carolina autosomal dominant ataxia, is an autosomal dominant disorder described in two families from North Carolina (65) and characterized by recurrent episodes of vertigo and ataxia associated with ocular abnormalities (diplopia, deficient smooth pursuit, gaze-evoked nystagmus). Attacks typically last for hours and acetazolamide is not effective. The onset varies from the third to the sixth decade. Slowly progressive cerebellar ataxia has been recorded in some affected individuals. Linkage analysis excluded the loci for EA1 and EA2 (66). Although sharing some common features with EA3 and designation of EA4 was suggested at the time of the mapping of EA3 (64), some other features justify this entity: abnormal eye movements, including abnormal smooth pursuit, nystagmus, and abnormal vestibuloocular reflex. Interictal myokymia was absent and patients did respond to treatment with acetazolamide.

##### Episodic Ataxia Type 7

EA7 has been reported in one American 4-generation family with autosomal dominant inheritance. The seven affected members presented with exercise- and excitement-triggered episodes including EA, weakness, and slurred speech (67) lasting hours to days. Two affected family members reported vertigo during attacks. EA onset was before the age of 20 years and attack frequency ranged from monthly to yearly and tended to decrease with age. There were no interictal findings on neurologic examination. By genomewide linkage and haplotype analysis this distinct type could be linked to chromosome 19q13 (67).

#### Acquired Episodic Ataxias

Since this article has a focus on genetic forms of episodic ataxias, acquired EAs are only mentioned briefly. Some authors proposed that adult onset and short episodes are the most specific features for distinguishing “symptomatic” (i.e., in the context of an acquired neurological disease) from primary EAs (68). First descriptions come from Multiple Sclerosis patients. Hereby, it is postulated that midbrain lesions may lead to interruption of the cerebello–thalamo–cortical pathway with subsequent induction of parietal diaschisis manifesting paroxysmal movement disorders, such as EA (69). Other causing diseases are Bickerstaff's-like brainstem encephalitis (70), stroke (71), Behcet's disease (72), paraneoplastic limbic encephalitis (e.g., Anti-Hu), and anti–contactin associated protein-like 2 (CASPR2) antibody-related autoimmune limbic encephalitis presenting with amnesia and seizures (73, 74). Anti-Hu-associated paraneoplastic limbic encephalitis presenting with EA and behavioral changes evolving to intractable epilepsy has been reported (75).

## Discussion

Reviewing treatment efforts of EA1 patients show that less than half of the reported patients responded to acetazolamide (AZA) treatment but also revealed that the majority of patients responded to different SCBs, in particular carbamazepine (CBZ) and phenytoin (PHT; Table 1; Figure 2). For all other drugs, no reasonable statements can be made due to low numbers (≤3 treatment trials). Unfortunately, the small size of reported treatment responses is a major limitation of this study, which emphasizes even more the necessity to review the known facts and develop effective treatment approaches. While for PHT only five patient reports were available of which three showed beneficial effects, seven responses to CBZ and two reports without clinical improvement could be found including one patient treated with only a minimum dose (400 mg/d). It is worth pointing out that the therapeutic response is variable even among individuals with the same genotype. Whether symptoms will be improved by a given drug can therefore not be fully predicted based on the expressed variant and associated biophysical alterations of channel function as genetic background or environmental factors may play important roles. Interestingly, a study of monozygotic twins with EA1 demonstrated inhomogeneous disease severity among the siblings thereby highlighting the impact of non-genetic factors (76).

In this study, we predict decreased rheobases and AUC in all but 3 *KCNA1* variants (I177N, V408A, and F414C) suggesting that LOF *KCNA1* variants generally induce an overall increase in neuronal excitability. The three variants with increased rheobases and AUC lack a complete electrophysiological characterization of the biophysical properties of the K_V_1.1 current (Table 1) and thus the simulated effects of these variants on neuronal firing must be interpreted with caution. Patients with these variants were not treated with CBZ and as such conclusions as to the benefit or potential harm of treatment of these patients with CBZ cannot be made.

Remarkably, biophysical dysfunction of K_V_1.1 channels in EA1 patients is almost exclusively based on depolarizing shifts of steady-state activation (Figure 3A), leading to LOF of *KCNA1* variant channels. Recently, Zhao et al. (77) predicted *in silico* that these depolarizing shifts broaden APs and interfere negatively with high frequency AP firing, suggesting that SCBs would be deleterious in EA1. However, the observed increase in neuronal excitability predicted by our models and the increased firing rates and decreased rheobases observed with K_V_1.1 channel block in sensory neurons (78), as well as clinical data reflecting positive outcomes of SCBs in EA1 patients (Table 1; Figure 2) provide a rational basis for the suggestion of the use of SCBs in EA1.

RLZ has been shown to inhibit neuronal firing across the CNS (23, 79–84), as well as inhibiting axonal firing (85). In a computational Purkinje cell model of ataxia exhibiting increased excitability, firing is returned to wildtype when multiple RLZ effects are taken into account (86), suggesting that the pleiotropy of RLZ is key to its efficacy. Additionally, RLZ decreases induced neuronal hyperexcitability in a number of animal disease models (87–89) with Hassani et al. (89) observing firing decreases in lesioned but not in unlesioned rats. The neuroprotective ability of RLZ to decrease hyperexcitability provides a further rationale for its potential in the treatment of EA1.

In our single compartment-based modeling presented here, predicted drug effects for riluzole are based on known mechanisms including reduction of persistent sodium channels, a notable shift of the steady-state inactivation and a reduced sodium conductance. However, distinctively to other SCBs in this study, RLZ also has the potential to reduce potassium conductance by dramatically slowing down the inactivation of K_V_1.4 channels within heterotetramers (16). In contrast to these reflected effects, this study neglects further additional mechanisms, such as an increase of calcium-activated potassium currents (90), an antagonism on NMDA receptors leading to reduced excitatory postsynaptic currents (80), and any effects of RLZ at the network level. In particular, RLZ partially inhibits high-voltage activated (HVA) calcium currents by up to 20% (91); however, this is specific to N and P/Q-type calcium channels (92), suggesting that RLZ inhibits calcium influx at presynaptic terminals. Although our single neuron simulations predict increased firing frequencies (AUC) as a result of RLZ, this may be mitigated in part at the network level through decreased synaptic transmission. Taking these and further additional mechanisms into account which are not reflected in our model, RLZ holds even greater potential in the treatment of EA1.

Our modeling-based findings for RLZ demonstrate similar effects on *KCNA1* variant firing to CBZ with increases in rheobase and AUC albeit with lower concentrations. Given the effectiveness of CBZ in an EA1 patient subset and the similarity of the effects of RLZ on firing rate, further investigation into the treatment of EA1 with RLZ is warranted. These findings would benefit from clinical data. Whereas, treatment reports of EA1 patients with RLZ are missing, RLZ has shown promise in a double-blind placebo-controlled trial in cerebellar ataxia patients with diverse ataxia etiologies in decreasing ataxia symptom severity with only mild adverse events reported (93). In a different double-blind placebo-controlled trial of RLZ in patients with spinocerebellar or Friedrich's ataxia, RLZ was also effective in treatment of the ataxias without severe adverse effects (94). Large sample size trials of RLZ in homogeneous forms of ataxia are needed to better understand the benefits of RLZ in ataxia and any potential ataxia-type specific efficacy (95, 96). However, the benefits of RLZ across diverse non-episodic ataxias suggest that treatment of common pathophysiological ataxia mechanisms such as altered neuronal firing may be beneficial in episodic ataxias.

Altogether, RLZ holds in our opinion great potential in the treatment of EA1, which needs certainly corroborated by further studies in heterologous expression systems and neurons.

## Data Availability Statement

The original contributions presented in the study are included in the Supplementary Material, further inquiries can be directed to the corresponding author.

## Author Contributions

SL, LS, and NK: designed and conceptualized study, acquisition of data, interpreted the data, and drafted and revised the manuscript for intellectual content. CB, UH, and YL: acquisition of data, interpreted the data, and drafted and revised the manuscript for intellectual content. NW, NS, and TW: interpreted the data and drafted and revised the manuscript for intellectual content. HL: interpreted the data, drafted and revised the manuscript for intellectual content, and study supervision. JB and JK: designed and conceptualized study, drafted and revised the manuscript for intellectual content, and study supervision. All authors approved the submitted version.

## Funding

This study was supported by the German Research Foundation in the frame of the Research Unit FOR-2715 (Grants Ko4877/3-1, Le1030/15-1, and He8155/1-1) and the German Federal Ministry for Education and Research (TreatION 01GM1907A). TW was supported by an intramural Clinician Scientist Fellowship granted by the Faculty of Medicine, University of Tübingen (419-0-0). We acknowledge support by Open Access Publishing Fund of University of Tübingen.

## Conflict of Interest

The authors declare that the research was conducted in the absence of any commercial or financial relationships that could be construed as a potential conflict of interest.

## Publisher's Note

All claims expressed in this article are solely those of the authors and do not necessarily represent those of their affiliated organizations, or those of the publisher, the editors and the reviewers. Any product that may be evaluated in this article, or claim that may be made by its manufacturer, is not guaranteed or endorsed by the publisher.
