# Supplementary material for: Therapeutic Potential of Sodium Channel Blockers as a Targeted Therapy Approach in KCNA1-Associated Episodic Ataxia and a Comprehensive Review of the Literature
Source: Front Neurol. 2021 Sep 9;12:703970. doi: 10.3389/fneur.2021.703970 (PMC8459024; doi:10.3389/fneur.2021.703970)
Supplement: Supplementary file 1 [file Data_Sheet_1.docx]

Supplementary Material

# Supplementary Figure Legend

**Localization of different mutations in the EAAT topology model** (Yernool et al., 2004). P.(Met128Arg) is located in transmembrane domain (TMD) 3, p.(Cys186Ser) in TMD 4b, p.(Pro290Arg) in TMD5, p.(Val393Iso) in TMD7 and p.(Arg499Glu) at the end of TMD8.
