# Supplementary figures and images for: Therapeutic Potential of Sodium Channel Blockers as a Targeted Therapy Approach in KCNA1-Associated Episodic Ataxia and a Comprehensive Review of the Literature
Source: Front Neurol. 2021 Sep 9;12:703970. doi: 10.3389/fneur.2021.703970 (PMC8459024; doi:10.3389/fneur.2021.703970)

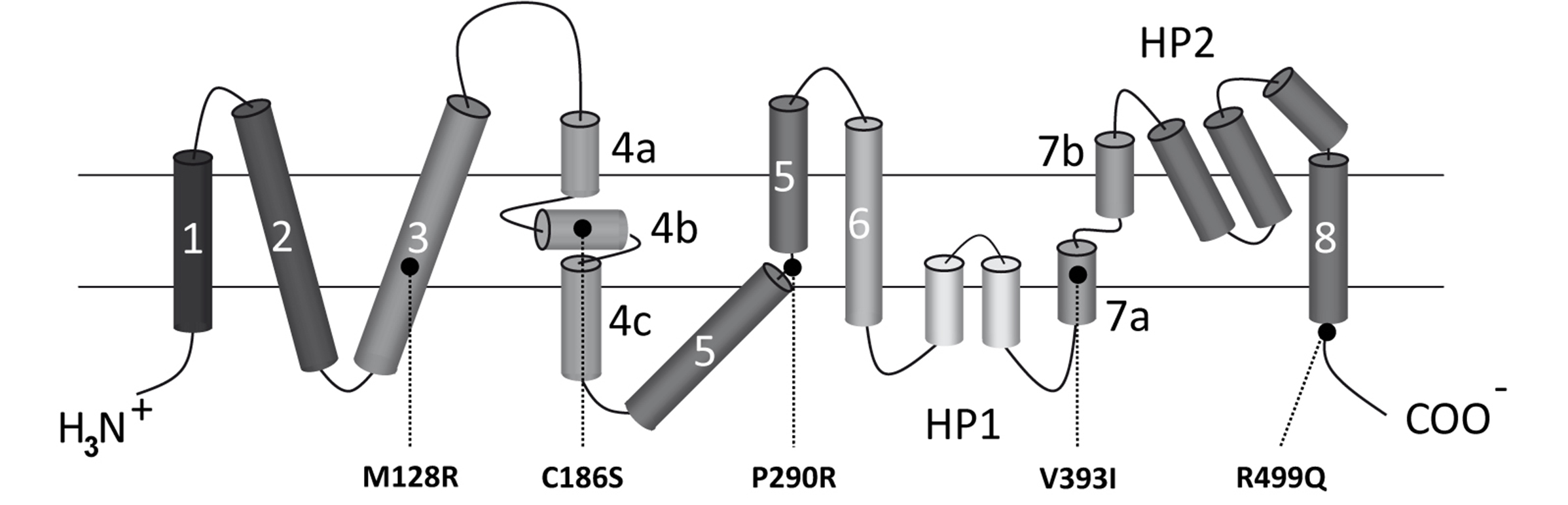

Supplement: Supplementary file 2 [file Image_1.JPEG]
